# Sex-specific disparities in postoperative adverse events following intracranial tumor surgery: insights from a tertiary neurosurgical center

**DOI:** 10.1007/s00701-025-06708-z

**Published:** 2025-11-13

**Authors:** Pavlina Lenga, Moritz Scherer, Philip Dao Trong, Sandro M. Krieg, Bogdana Suchorska

**Affiliations:** 1 Department of Neurosurgery, Heidelberg University Hospital, Im Neuenheimer Feld 400, 69120 Heidelberg, Germany; 2 Medical Faculty of Heidelberg, Heidelberg University, Heidelberg, Germany; 3 Department of Neurosurgery, Vivantes Neukölln, Berlin, Germany

**Keywords:** Adverse events, Neuro-oncology, Gender disparities, Tumor surgery

## Abstract

**Background:**

Growing evidence suggests that patient sex may influence perioperative outcomes in neurosurgery, yet the extent to which gender differences shape morbidity following intracranial tumor resection remains unclear. Elucidating these disparities is essential for refining risk stratification, tailoring perioperative management, and improving resource allocation in neuro-oncological practice.

**Methods:**

A prospective single-center observational study was performed between January 2023 and December 2023, enrolling all adult patients undergoing surgery for for **intracranial mass lesions (neoplasms and tumor-like non-neoplastic inflammatory lesions)**. Perioperative data, including demographic variables, tumor pathology, and adverse events (AEs) within 30 days of surgery, were recorded in a standardized database. The Clavien–Dindo classification was used to grade AEs. Logistic regression identified independent predictors of AEs, adjusting for age, sex, tumor location, and surgical urgency.

**Results:**

Among 1173 patients (mean age 57.4 ± 15.3 years; 500 men, 673 women), men more frequently had gliomas (38.8% vs. 20.4%), whereas women exhibited significantly higher rates of meningiomas (41.8% vs. 28.2%) and neurinomas (8.8% vs. 4.2%; p < 0.05). Overall, 149 patients (12.7%) experienced one or more AEs. Men displayed a slightly higher unadjusted AE rate (14.0% vs. 11.7%) and revision rate (5.8% vs. 3.0%) without statistical significance. Women, however, required unplanned ICU or IMC admission more often (22.1% vs. 17.4%, *p* = 0.047). In the multivariable model, older age (*p* = 0.004), infratentorial tumor location (*p* = 0.017), and emergency surgery (*p* = 0.002) were independent risk factors for th occurrence of AE, while sex was not among the registered AEs.

**Conclusions:**

These findings highlight sex‐specific differences in tumor distribution and postoperative outcomes in intracranial tumor surgery. Women were more likely to require escalated postoperative care, such as ICU or IMC admission, whereas men exhibited a higher crude rate of complications and revision surgeries. However, after adjusting for confounders such as age, tumor location, and surgical urgency, male sex was associated with a modestly reduced risk of adverse events, emphasizing the complex interplay of biological, clinical, and systemic factors in perioperative outcomes. Understanding these sex‐specific patterns is crucial for tailoring perioperative care strategies, improving patient outcomes, and advancing individualized treatment protocols in neuro-oncology. Further research should explore the underlying mechanisms driving these disparities to inform evidence-based, gender-sensitive neurosurgical care.

**Supplementary Information:**

The online version contains supplementary material available at 10.1007/s00701-025-06708-z.

## Introduction

Gender disparities in healthcare outcomes have received growing attention across multiple medical disciplines, including neurosurgery [1, 17, 28]. Although studies have identified potential differences in morbidity, mortality, and functional recovery after various neurosurgical procedures, the specific impact of patient gender on postoperative adverse events (AEs) following intracranial tumor surgery remains incompletely understood. Several retrospective investigations have suggested that female and male patients may experience distinct postoperative courses, citing variations in tumor biology, hormonal influences, immune responses, and healthcare-seeking behavior as possible underlying factors [6, 22, 23]. However, these observations require further clarification, particularly through prospective analyses that can minimize selection bias and ensure standardized data collection.

Biological and hormonal mechanisms are frequently posited to contribute to sex-related differences in oncological behavior and surgical outcomes. For example, certain intracranial tumors, such as meningiomas and vestibular schwannomas, are known to exhibit hormone receptor expression that may promote or modulate tumorigenesis, potentially affecting surgical difficulty and recovery [24]. In contrast, malignant gliomas, which are more common in males, may involve disparate gene expression profiles and immune microenvironments that influence tumor aggressiveness and patient resilience in the perioperative period [23, 27].

From a clinical standpoint, elucidating these gender-based differences in adverse events is essential for improving risk stratification, guiding informed consent, and optimizing perioperative care pathways. Moreover, given the complexity and cost-intensive nature of neurosurgical procedures for intracranial tumors, understanding how patient sex influences complications may assist in targeted resource allocation and multidisciplinary care planning. Despite these recognized needs, few prospective studies have systematically captured and analyzed sex-specific data related to AEs in the intracranial tumor population.

Against this background, we established a prospective institutional database encompassing all patients undergoing intracranial tumor surgery at our center. This standardized data repository enables robust, real-time capture of perioperative details, patient characteristics, and postoperative complications, thereby providing a unique opportunity to explore the relationship between gender and adverse events. In this paper, we present our findings regarding the incidence, nature, and potential predictors of AEs in male versus female patients.

## Methods

### Study design and ethics

A prospective, single-center observational study was conducted at a tertiary care academic hospital between January 2023 and December 2023. All aspects of the research followed the principles outlined in the Declaration of Helsinki and were approved by the local Institutional Review Board (reference number S-425/2022). Written informed consent was waived as the documentation of adverse events (AEs) is an integral component of the institution’s routine clinical documentation and quality assurance processes. Consecutive adult patients (≥ 18 years) admitted for intracranial tumor resection defined as space-occupying neoplasms and tumor-like non-neoplastic inflammatory lesions, during the specified study period were included. Exclusion criteria encompassed (1) prior neurosurgical interventions within the previous 30 days, (2) incomplete postoperative follow-up data. Both elective and emergency cases were eligible, provided the primary surgical goal was tumor removal, regardless of histopathological subtype.

### Perioperative management

All patients received standardized perioperative care.

All patients were managed under departmental standard operating procedures (SOPs) that were in place throughout the study period. These SOPs specify (i) structured preoperative assessment and optimization; (ii) standardized antibiotic, anticoagulation, steroid, and antiseizure prophylaxis policies; (iii) indications for intraoperative adjuncts (neuronavigation, intraoperative monitoring, awake mapping when appropriate); (iv) extubation and immediate postoperative neurological surveillance; (v) criteria-based triage to ICU/IMC versus normal ward; (vi) early postoperative imaging; (vii) mobilization and venous-thromboembolism (VTE) prevention; and (viii) the prospective AE capture process (POPAE form at discharge, senior review, 30-day readmission flagging, and presentation at morbidity & mortality conferences) [5, 19, 20]. The SOP components are summarized in Supplementary Table S1 and align with our previously published AE program and intracranial tumor cohort methodology.

### Data collection and adverse event monitoring

Demographic, clinical, and surgical data were prospectively entered into a secure database by both board-certified neurosurgeons and neurosurgical residents, as previously described [5]. Postoperative adverse events (AEs) occurring within the first 30 days, including but not limited to wound infection, hematoma, new neurological deficits, and thromboembolic events, were meticulously documented. Unscheduled readmissions triggered an automatic notification for the neurosurgical team to facilitate prompt evaluation. Ambiguous or complex cases were reviewed in morbidity and mortality (M&M) conferences, ensuring consistency in AE classification. Each data entry was cross-referenced by a senior neurosurgeon to minimize transcription errors, and any discrepancies were resolved by consensus during departmental M&M conferences. To ensure completeness, a final audit was conducted after the 30-day follow-up window closed for every enrolled patient. The Clavien–Dindo classification was deployed to provide the gravity of single adverse events [8]. **Surgical goal not achieved.** Before incision, the lead surgeon records the intended goal (gross-total resection; planned subtotal/partial resection for decompression; or diagnostic biopsy). “Surgical goal not achieved” was coded when the postoperative result deviated from this intention for non-planned reasons and prompted additional treatment within 30 days. Examples include failure to reach intended GTR with clinically relevant residual on early imaging; insufficient decompression with persistent mass effect/hydrocephalus requiring medical escalation or CSF diversion; or nondiagnostic biopsy requiring repeat sampling. If the goal was intentionally revised intraoperatively for safety and documented accordingly, the case was not labeled as “goal not achieved.” In the Clavien–Dindo framework, events managed non-operatively were graded **2**, whereas events requiring unplanned surgical/endoscopic/radiological intervention were graded **3**.

### Statistical analysis

Statistical analyses were conducted using SPSS (version 26.0.0.0, IBM, Armonk, NY, USA) and R (IBM Corp., Armonk, NY, USA). Categorical variables were summarized as frequencies and percentages, while continuous variables were presented as means ± standard deviations or medians with interquartile ranges, based on distribution. Group comparisons for categorical parameters employed the chi-square or Fisher’s exact test, as appropriate. Continuous variables were evaluated using Student’s t-test or the Mann–Whitney U test. Logistic regression models were constructed to identify independent predictors of postoperative adverse events, adjusting for clinically relevant confounders. All reported p-values were two-sided, with a significance threshold set at p < 0.05.

## Results

### Study population and baseline characteristics

Between January 2023 and December 2023, a total of 1173 patients (500 men and 673 women) underwent surgical treatment for intracranial tumors, with a mean age of 57.4 ± 15.3 years (range 18–93). Most procedures (93.4%) took place under elective conditions, whereas 6.6% (78/1173) were emergencies. Meningioma (31.1%) and glioma (28.2%) were the most frequent pathologies in the overall cohort. A pronounced sex difference in tumor type was observed, with gliomas occurring in 194 of the 500 men (38.8%) but in only 137 of the 673 women (20.4%), while meningiomas predominated in 281 of the 673 women (41.8%) compared to 141 of the 500 men (28.2%). Neurinomas followed a similar pattern, appearing in 8.8% of women and 4.2% of men. Detailed demographic and clinical data are presented in Table 1.

**Table 1:** Baseline characteristics by sex

	Overall *N* = 1173	Male (*n* = 500)	Female (*n* = 673)	p
Age, years (mean, SD)	57.4 (15.3)	57.9 (14.3)	56.4 (11.2)	0.062
Non-Elective	78 (6.6%)	33 (6.6%)	45 (6.7%)	0.953
Elective	1095 (93.4%)	467 (93.4%)	628 (93.3%)	
Supratentorial	515 (43.9%)	277 (55.4%)	238 (35.4%)	**0.001**
Infratentorial	658 (56.1%)	223 (44.6%)	435 (64.6%)	
Pathology				** < 0.001**
Glioma	331 (28.2%)	194 (38.8%)	137 (20.4%)	
Meningioma	365 (31.1%)	84 (16.8%)	281(41.8%)	
Ependymoma	11 (0.9%)	8 (1.6%)	3 (0.4%)	
Neurinoma Schwannoma (neurinoma)	58 (4.9%)	30 (6.0%)	28 (4.2%)	
Pituitary adenoma	95 (8.1%)	49 (9.8%)	46 (6.8%)	
Pineal region tumors	6 (0.5%)	1 (0.2%)	5 (0.7%)	
Metastasis	204 (17.4%)	91 (18.2%)	113 (16.8%)	
Hemangioblastoma	15 (1.3%)	5 (1.0%)	10 (1.5%)	
Sarcoma	10 (0.9%)	3 (0.6%)	7 (1.0%)	
Chordoma	1 (0.1%)	1 (0.2%)	0 (0.0%)	
Epidermoid tumor	9 (0.8%)	3 (0.6%)	6 (0.9%)	
Embryonal tumor	7 (0.6%)	6 (1.2%)	1 (0.1%)	
Inflammation Inflammatory/tumor-like lesion	42 (3.6%)	25 (5.0%)	17 (2.5%)	

Values are n (%) unless otherwise specified. Age is presented as mean ± SD (years). Percentages in the Male and Female columns are column percentages; the Overall column uses *N* = 1,173. p-values compare Male vs Female using Pearson’s χ^2^ test (or Fisher’s exact test when expected counts < 5) for categorical variables and an unpaired t-test (or Mann–Whitney U for non-normal distributions) for age. For the Pathology block, the p-value reflects a global χ^2^ test across all pathology categories. Abbreviations: *ICU*, intensive care unit; *IMC*, intermediate care unit; *SD*, standard deviation; schwannoma (neurinoma) used synonymously

### Incidence and distribution of postoperative adverse events

Overall, 149 of the 1173 patients (12.7%) experienced at least one surgery-related adverse event (AE). Of these, 70 were men (14.0% of all male patients) and 79 were women (11.7% of all female patients). Revision procedures were required in 49 cases (4.2% overall), corresponding to 29 men (5.8% of all men) and 20 women (3.0% of all women). Although men had a numerically higher crude AE rate and a higher rate of revision surgery, neither difference reached statistical significance. The distribution of AEs according to Clavien–Dindo classification is illustrated in Table 2.

**Table 2:** Postoperative adverse events by Clavien–Dindo grade and sex

CDC *	Overall *N* = 1173	Male (*n* = 500)	Female (*n* = 673)	p
Wound Event				0.066
Grade 2	4 (0.3%)	1 (0.2%)	3 (0.4%)	
Grade 3	11 (0.9%)	8 (1.6%)	3 (0.4%)	
Dural leak				0.598
Grade 2	9 (0.8%)	4 (0.8%)	5 (0.7%)	
Grade 3	9 (0.8%)	3 (0.6%)	6 (0.9%)	
Rebleeding				0.667
Grade 2	4 (0.3%)	0 (0.0%)	4 (0.6%)	
Grade 3	8 (0.7%)	3 (0.6%)	5 (0.7%)	
Grade 4	7 (0.6%)	3 (0.6%)	4 (0.6%)	
Grade 5	1 (0.1%)	0 (0.0%)	1 (0.1%)	
New neurological deficit				0.789
Grade 1	58 (4.9%)	25 (5.0%)	33 (4.9%)	
Grade 2	38 (3.2%)	18 (3.6%)	20 (3.0%)	
Grade 3	7 (0.6%)	1 (0.2%)	6 (0.9%)	
Grade 4	3 (0.3%)	0 (0.0%)	3 (0.4%)	
Surgical goal not achieved				0.116
Grade 2	3 (0.3%)	0 (0.0%)	3 (0.4%)	
Grade 3	1 (0.1%)	1(0.2%)	0 (0.0%)	

Values are n (%). Percentages in the Male and Female columns are column percentages; the Overall column uses *N* = 1,173. p-values compare Male vs Female across grades within each domain (global test). Clavien–Dindo grades: 1 = deviation from normal course with minor intervention; 2 = pharmacological treatment; 3 = surgical/endoscopic/radiological intervention; 4 = life-threatening complication requiring ICU; 5 = death. Abbreviation: CDC, Clavien–Dindo classification

#### Wound events

Wound-related complications arose in patients across multiple pathologies but were most frequent in those with gliomas and meningiomas. Among the 7 glioma patients who developed wound events, 4 were men and 3 were women; 6 of these individuals required revision. In the meningioma subgroup, 3 patients (1 man and 2 women) exhibited wound complications, and all underwent surgical revision. Wound issues were also encountered in 3 patients with metastatic lesions (1 man and 2 women, with 2 requiring revision) and in isolated cases of ependymoma and hemangioblastoma.

#### Dural leaks

A total of 11 patients presented with postoperative cerebrospinal fluid (CSF) leakage. Seven of these occurred in the glioma group (5 men and 2 women), where 2 required revision. Four dural leaks were documented in patients with neurinomas (1 man and 3 women), and half of these required reoperation or reinforcement procedures. A summary of dural leak incidents by sex and tumor type appears in Table 3.

**Table 3:** Summary of surgery-related adverse events

	Overall *N* = 1173	Male (*n* = 500)	Female (*n* = 673)	**p**	Revision surgery Overall *N* = 1173	Male *N* = 500	Female *N* = 673	p
Wound event				0.677				0.569
Glioma	7 (2.1%)	6 (1.2%)	7 (1.0%)		6 (1.8%)	4 (0.8%)	2 (0.3%)	
Meningioma	3 (0.8%)	2 (0.4%)	1 (0.1%)		3 (0.8%)	3 (0.6%)	0 (0.0%)	
Ependymoma	1 (9.1%)	1 (0.2%)	0 (0.0%)		0 (0.0%)	0 (0.0%)	0 (0.0%)	
Metastasis	3 (1.5%)	2 (0.4%)	1 (0.1%)		2 (1.0%)	2 (0.4%)	0 (0.0%)	
Hemangioblastoma	1 (6.7%)	1 (0.2%)	0 (0.0%)		1 (6.7%)	1 (0.2%)	0 (0.0%)	
Dural leak				0.789				0.777
Glioma	7 (2.1%)	5 (1.0%)	2 (0.3%)		2 (0.6%)	1 (0.2%)	1 (0.1%)	
Meningioma	2 (0.5%)	0 (0.0%)	2 (0.3%)		2 (0.5%)	0 (0.0%)	2 (0.3%)	
Ependymoma	1 (9.1%)	1 (0.2%)	0 (0.0%)		0 (0.0%)	0 (0.0%)	0 (0.0%)	
Schwannoma (neurinoma)	4 (6.9%)	2 (0.4%)	2 (0.3%)		2 (3.4%)	2 (0.4%)	0 (0.0%)	
Pituitary adenoma	1 (10.5%)	1 (0.2%)	0 (0.0%)		0 (0.0%)	0 (0.0%)	0 (0.0%)	
Pineal region tumor	1 (16.7%)	1 (0.2%)	0 (0.0%)		1 (16.7%)	1 (0.2%)	0 (0.0%)	
Metastasis	1 (1.0%)	0 (0.0%)	0 (0.0%)		1 (1.0%)	0 (0.0%)	1 (0.1%)	
Hemangioblastoma	1 (6.7%)	0 (0.0%)	1 (0.1%)		1 (6.7%)	0 (0.0%)	1 (0.1%)	
Rebleeding				0.321				0.465
Glioma	10 (3.0%)	4 (0.8%)	6 (0.9%)		8 (2.4%)	4 (0.8%)	4 (0.6%)	
Meningioma	4 (1.1%)	1 (0.2%)	3 (0.5%)		3 (0.8%)	1 (0.2%)	2 (0.3%)	
Schwannoma (neurinoma)	3 (5.2%)	2 (0.4%)	1 (0.1%)		3 (5.2%)	2 (0.4%)	1 (0.1%)	
Metastasis	2 (1.1%)	1 (0.2%)	1 (0.1%)		2 (1.1%)	1 (0.2%)	1 (0.1%)	
Inflammatory/tumor-like lesion	1 (7.1%)	1 (0.2%)	0 (0.0%)		1 (7.1%)	1 (0.2%)	0 (0.0%)	
Surgical goal not achieved				0.479				0.789
Meningioma	1 (0.3%)	1 (0.2%)	0 (0.0%)		1 (0.3%)	1 (0.2%)	0 (0.0%)	
Pituitary adenoma	1 (10.5%)	1 (0.2%)	0 (0.0%)		0 (0.0%)	0 (0.0%)	0 (0.0%)	
Metastasis	1 (1.0%)	0 (0.0%)	0 (0.0%)		0 (0.0%)	0 (0.0%)	0 (0.0%)	
Epidermoid tumor	1 (11.1%)	1 (0.2%)	0 (0.0%)		0 (0.0%)	0 (0.0%)	0 (0.0%)	
New neurological deficit				0.511				0.321
Glioma	34 (10.3%)	18 (3.6%)	16 (2.4%)		1 (0.3%)	0 (0.0%)	0 (0.0%)	
Meningioma	42 (11.5%)	21 (4.2%)	21 (3.1%)		3 (0.8%)	0 (0.0%)	3 (0.5%)	
Schwannoma (neurinoma)	9 (15.5%)	5 (1.0%)	4 (0.5%)		2 (3.4%)	2 (0.2%)	2 (0.3%)	
Pituitary adenoma	3 (3.2%)	2 (0.4%)	1 (0.1%)		0 (0.0%)	0 (0.0%)	0 (0.0%)	
Pineal region tumor	1 (16.7%)	1 (0.2%)	0 (0.0%)		0 (0.0%)	0 (0.0%)	0 (0.0%)	
Metastasis	2 (1.1%)	1 (0.2%)	1 (0.1%)		2 (1.1%)	1 (0.1%)	1 (0.1%)	
Hemangioblastoma	1 (6.7%)	1 (0.2%)	0 (0.0%)		1 (6.7%)	1 (0.1%)	0 (0.0%)	
Embryonal tumor	1 (14.3%)	1 (0.2%)	0 (0.0%)		1 (14.3%)	1 (0.1%)	0 (0.0%)	
Secondary transfer to IMC or ICU				0.477				
Glioma	12 (3.6%)	8 (1.6%)	4 (0.6%)		––	––	––	––
Meningioma	10 (2.7%)	5 (1.0%)	5 (0.8%)		––	––	––	––
Schwannoma (neurinoma)	2 (3.5%)	1 (0.2%)	1 (0.1%)		––	––	––	––
Pituitary adenoma	4 (4.2%)	3 (0.6%)	(0.1%)		––	––	––	––
Pineal region tumor	1 (6.7%)	1 (0.2%)	0 (0.0%)		––	––	––	––
Metastasis	9 (4.4%)	6 (1.2%)	3 (0.5%)		––	––	––	––
Hemangioblastoma	2 (13.3%)	1 (0.2%)	(0.1%)		––	––	––	––
Sarcoma	1 (10.0%)	1 (0.2%)	0 (0.0%)		––	––	––	––
Epidermoid tumor	1 (11.1%)	1 (0.2%)	0 (0.0%)		––	––	––	––
Embryonal tumor	1 (14.3%)	1 (0.2%)	0 (0.0%)		––	––	––	––
Inflammatory/tumor-like lesion	3 (7.1%)	2 (0.4%)	(0.1%)		––	––	––	––
Death				0.788				
Supratentorial	3 (0.6%)	2 (0.4%)	1 (0.1%)		–––-	–––-	–––-	–––-
Infratentorial	2 (0.3%)	1 (0.2%)	1 (0.1%)		–––-	–––-	–––-	–––-

All data are presented as numbers (n) and percentages unless otherwise specified

#### Postoperative hemorrhage

Hemorrhagic complications were reported in 10 glioma patients (6 men and 4 women) and resulted in 8 revisions; meningioma cases accounted for 4 postoperative hemorrhages (1 man and 3 women), 3 of which required reoperation. Among patients with neurinomas, 3 individuals (2 men and 1 woman) experienced postoperative bleeding, and all underwent surgical evacuation. Two patients (1 man and 1 woman) with metastatic tumors and one patient (male) with an inflammatory lesion also required revision due to hemorrhage. Further details on the incidence and revision rates for each histological subgroup are provided in Table 2.

#### New neurological deficits

In the immediate postoperative period, 34 patients with glioma (18 men and 16 women) developed new neurological deficits, and 1 of these cases underwent surgical exploration of the resection cavity. Among meningioma patients, 42 individuals (21 men and 21 women) presented with new deficits, and again only 1 ultimately required reoperation. Although these neurological complications were more numerous than some other specific AEs, most cases were managed conservatively or with additional supportive measures.

All data is presented by Table 3.

### Secondary admissions to ICU or IMC and mortality

A total of 236 patients (20.1%) required unplanned admission to an Intensive Care Unit (ICU) or Intermediate Care (IMC) unit due to postoperative concerns. Women were more frequently transferred to higher-level care than men (149/673, 22.1% vs. 87/500, 17.4%, *p* = 0.047). The overall mortality in the cohort was 0.8% (5/1173), comprising 3 men and 2 women, with no statistically significant difference between the sexes. Causes of death included massive intracranial hemorrhage (*n* = 1), pulmonary embolism (*n* = 2), and rapidly progressing tumor burden (*n* = 2).

### Multivariable regression analysis

A logistic regression model evaluated the independent impact of sex, age, urgency of surgery, and tumor location on the occurrence of any postoperative AE. Emergency surgery, infratentorial tumor location, and advancing age emerged as significant predictors of higher AE risk. Male sex was associated with a modest reduction in the likelihood of sustaining an AE (OR = 0.79, 95% CI 0.62–0.99, *p* = 0.042), indicating that the slight excess of AEs in men observed in unadjusted analyses was mitigated once these factors were considered (Table 4).

**Table 4:** Multivariable logistic regression for 30-day adverse events

Risk factor	OR (95% CI)	*p*-value
Age	1.2 (1.1–2.3)	**0.004**
Sex (male)	0.7 (0.5–0.9)	0.712
Infratentorial location	1.8 (1.1–5.1)	**0.017**
Emergency surgery	2.1 (1.5–2.7)	**0.002**

Shown are adjusted odds ratios (OR) with 95% confidence intervals (CI) and p-values from the model adjusting for age, sex, tumor location, and surgical urgency

## Discussion

Neurosurgical interventions pose substantial challenges and are frequently associated with high morbidity and mortality. However, current evidence regarding potential gender disparities in outcomes following intracranial tumor surgery remains limited. Here, we analyzed 1,173 consecutive cases of intracranial tumor surgery to explore possible sex-related differences in the incidence of postoperative adverse events (AEs) within the first 30 days after surgery. Of these patients, 673 were women and 500 were men, with men more commonly presenting with gliomas, while women predominantly harbored meningiomas or vestibular schwannomas. Despite these histopathological variations, there were no significant sex-based discrepancies in overall complication rates (14.0% vs. 14.2%; *p* = 0.973), revision surgeries (5.8% vs. 3.1%), or 30-day mortality (~ 0.40% vs. ~ 0.44%). Crucially, multivariable logistic regression—controlling for age, emergent status, and tumor type—showed that sex was not an independent predictor of postoperative AEs (*p* = 0.973), whereas advanced age and emergent surgery were significantly linked to higher complication rates.

Our overall complication rate of 14.1% (166/1,173) aligns with the 12–20% range reported across neurosurgical oncology cohorts [6, 9, 15]. Notably, men (14.0%) and women (14.2%) in our series exhibited almost identical incidences of AEs, paralleling the findings of Dasenbrock et al., who analyzed 51,000 craniotomies for tumor and found an in-hospital complication rate of approximately 15–17%. Although their unadjusted analyses suggested that female patients experienced longer hospital stays than males, these differences failed to persist in the multivariable model once factors such as age, comorbidity burden, emergent status, and tumor type were incorporated. Similarly, our logistic regression model identified advanced age and emergency surgery—rather than sex—as principal predictors of postoperative morbidity, implying that clinical acuity and physiologic reserve overshadow any modest influence of biological sex on near-term surgical risk. Comparable observations have been noted in other large-scale studies [3, 11], collectively reinforcing the notion that sex alone is rarely an independent driver of short-term neurosurgical outcomes when robust statistical adjustments are made. Despite drawing primarily on retrospective data, these large administrative studies do offer considerable breadth and a diverse patient population. Nevertheless, they are inherently subject to potential inaccuracies in coding, variances in outcome definitions, and residual confounding not captured by billing data [26]. While this methodological rigor enhances internal validity, it also raises questions about external generalizability, as other institutions—particularly those with smaller volumes or fewer specialized resources—might record more pronounced sex-related outcomes. Confirmatory studies across diverse practice environments and with extended follow-up intervals are warranted to determine whether these observations hold true for long-term functional status, tumor recurrence, or quality-of-life metrics, where sex-linked physiological and hormonal influences could yet play a more significant role [14, 30].

In our cohort, revision procedures occurred in 3.1% of female patients versus 5.8% of male patients, reflecting a modest but statistically nonsignificant difference (*p* > 0.05). These figures fall within the spectrum reported in large administrative and single-center analyses; for instance, Brandi et al. found reoperation rates ranging from 4 to 6% across various tumor pathologies in a nationwide dataset [3]. Although higher revision rates among men have been hypothesized—often in the setting of aggressive gliomas or residual tumor—once age, comorbidities, and emergent status are controlled, the impact of sex on reoperation risk typically diminishes [6, 7, 13, 21]. Our findings, therefore, corroborate the notion that clinical factors such as tumor aggressiveness, acuity of presentation, and baseline frailty overshadow biological sex in determining the likelihood of early revision. Furthermore, the overall perioperative mortality in our series (~ 0.43% combined) is notably low, aligning more closely with centers that demonstrate robust perioperative protocols, advanced surgical techniques, and high institutional volume [14, 25, 30]. While the Nationwide Inpatient Sample often cites 1–3% mortality for intracranial tumor resections [6, 9, 15], variations in patient selection, hospital resources, and referral patterns can influence these outcomes. Crucially, we found no sex-specific difference in mortality (~ 0.40% in men vs. ~ 0.44% in women), indicating that, under standardized perioperative conditions, sex alone is unlikely to be a determinant of short-term survival.

In our series, female patients had a notably higher rate of ICU admissions than males (149 vs. 87; *p* = 0.047), even though sex did not emerge as an independent risk factor for postoperative adverse events. Similar discrepancies in ICU utilization by sex have been documented in observational cohorts, where healthcare providers may adopt more vigilant monitoring strategies for women, or female patients may report symptoms more frequently or intensely [18, 29]. However, neither approach nor perception translates into worse short-term outcomes in our data, underscoring that ICU admission patterns may reflect institutional practices or clinician caution rather than intrinsic biological risk. Instead, advanced age and emergency surgery stood out as the principal drivers of AEs, echoing findings from large-scale analyses such as the Nationwide Inpatient Sample (NIS), where urgent admissions and older patient cohorts consistently exhibited higher complication rates [2, 6, 7]. These results highlight how clinical urgency and physiologic reserve can overshadow smaller sex-related differences in perioperative risk. Notably, our prospective single-center design, characterized by real-time Morbidity and Mortality (M&M) reviews and standardized event reporting, likely provides more precise adverse event capture than retrospective or administrative database studies. Although this approach bolsters internal validity, the absence of detailed comorbidity data limits our ability to control for preexisting health conditions in assessing ICU admissions or complications.

Our data highlight pronounced sex-based disparities in tumor distribution: gliomas were identified in 38.8% of men but only 20.4% of women, with glioblastoma specifically occurring in 24.8% of male patients versus 2.4% of females. Conversely, meningiomas appeared in 41.8% of women compared to 16.8% of men. These findings align closely with large-scale epidemiological reports, including the CBTRUS Statistical Report, which confirm that high-grade gliomas (e.g., glioblastoma) are more common in males, whereas meningiomas show a female preponderance often exceeding a 2:1 ratio [23, 31]. Proposed mechanisms include sex hormone receptor expression (particularly progesterone receptors) in many meningiomas, in contrast to the molecular pathways—such as RB inactivation and immune modulation—implicated in aggressive gliomas favoring male patients [27]. Moreover, recent systematic reviews of vestibular schwannomas (also more prevalent in females) suggest hormonal influences may contribute to tumorigenesis or growth, though the precise pathophysiological links remain incompletely characterized [16].

Notably, we observed a significantly higher rate of infratentorial lesions among female patients, which also emerged as a risk factor for postoperative AEs. This outcome is consistent with multiple neurosurgical studies suggesting that posterior fossa tumors carry an inherently higher risk of complications due to the tight anatomical confines of the cerebellopontine angle (CPA) or other infratentorial compartments [12, 16]. Even small postoperative hematomas, edema, or cranial nerve insults can cause disproportionately severe symptoms (e.g., obstructive hydrocephalus, brainstem compression), elevating morbidity. Because women are overrepresented among certain infratentorial tumor types—particularly vestibular schwannomas and posterior fossa meningiomas [4, 10]—they may face a heightened baseline risk of mechanical or compressive complications. Although our multivariable analysis did not identify sex per se as a predictor of adverse outcomes, the interplay between female sex and infratentorial location raises the possibility that, in some subsets of women, tumor anatomy and local mass effect outstrip any protective or risk-enhancing biological factors.

### Study limitations and strengths

While this single-center, prospective design provides a high level of detail on adverse events within the first 30 days—a critical period frequently used as a benchmark for surgical quality—several factors may influence the extrapolation of our results. Pathology heterogeneity is a limitation. We deliberately included non‑neoplastic inflammatory tumor‑like lesions to reflect real‑world intracranial mass‑lesion surgery and to capture sex‑related perioperative risk that may transcend histology. While this improves generalizability, it may dilute tumor‑specific effects and leaves residual confounding by pathology; therefore, our findings should be interpreted as pertaining to surgery for intracranial mass lesions rather than any single tumor entity. We did not collect standardized subcategories of neurological deficits (motor, sensory, cranial-nerve, language, cerebellar, etc.) in the registry; the endpoint was captured as a composite “new neurological deficit.” As a result, sex-specific patterns within deficit subtypes could not be assessed, and future iterations of our prospective program will include structured subtype fields to enable more granular analyses. We did not capture a **standardized primary reason** for each IMC/ICU transfer; multiple triggers often co-existed (neurological observation, respiratory/hemodynamic support, or management of complications). The rigorous focus on immediate postoperative outcomes necessarily narrows the window for capturing potential later sequelae, such as tumor recurrence or long-term functional decline. Additionally, comorbidities were not systematically quantified, limiting our capacity to fully adjust for underlying health conditions that could predispose patients to adverse events. These considerations partially constrain the generalizability of our findings to centers with different patient profiles, practice patterns, or resource availabilities. Nonetheless, the strengths of this investigation remain considerable. By documenting postoperative complications in real time, with weekly Morbidity and Mortality (M&M) reviews, we achieve a level of accuracy surpassing that of many retrospective or registry-based analyses, where underreporting or coding discrepancies can obscure outcome patterns. Moreover, the uniform application of standardized perioperative pathways allows consistent care across tumor subtypes and patient subgroups, enhancing the internal validity of our conclusions regarding potential sex-based disparities. Consequently, although a broader multicenter approach and extended follow-up might further enrich our understanding, the design of this study—centered on 30-day adverse event monitoring—offers a robust foundation for examining short-term neurosurgical risks and provides valuable insights into the clinical and biological factors influencing early postoperative courses.

## Conclusions

This prospective single-center study of 673 female and 500 male patients undergoing intracranial tumor resection reveals no significant sex-based disparity in short-term postoperative outcomes, despite men more frequently harboring gliomas and women exhibiting higher ICU admissions and infratentorial lesions. Advanced age and emergent surgery—rather than sex—emerged as the key risk factors for adverse events, underscoring that clinical acuity and physiological reserve exert a stronger influence on immediate complications than biological sex alone. By employing standardized perioperative protocols and systematically tracking 30-day complications, our work confirms the feasibility of mitigating subtle sex-linked variations in the short term.

## Supplementary Information

Below is the link to the electronic supplementary material.

ESM 1 DOCX (18.1 KB)

## Data Availability

The datasets generated during and/or analyzed during the current study are available from the corresponding author on reasonable request.
