# Supplementary material for: Sex-specific disparities in postoperative adverse events following intracranial tumor surgery: insights from a tertiary neurosurgical center
Source: Acta Neurochir (Wien). 2025 Nov 13;167(1):292. doi: 10.1007/s00701-025-06708-z (PMC12615520; doi:10.1007/s00701-025-06708-z)
Supplement: Supplementary file 1 — DOCX (18.1 KB) [file 701_2025_6708_MOESM1_ESM.docx]

**Supplementary Table S1. Standardized perioperative pathway for intracranial mass‑lesion surgery at our center (SOPs constant during study period)**

| **Domain** | **Standard (concise)** | **Notes / thresholds** |
| --- | --- | --- |
| **Preoperative assessment** | Structured history/exam; ASA class; cardiopulmonary risk review; medication reconciliation (anticoagulants/antiplatelets); labs incl. CBC, electrolytes, coagulation; MRI/CT within institutional recency windows; anesthesiology pre‑assessment for high‑risk cases. | Elective cases: optimization before surgery when feasible. Emergency cases: abbreviated pathway with documentation of deviations. |
| **Antibiotic prophylaxis** | Single pre‑incision dose of a first‑line cephalosporin (weight‑adjusted; within 60 min before incision); re‑dose per duration/EBL; allergy alternatives per SOP. | No routine post‑op continuation in clean cases. |
| **Antiseizure management** | Pre‑op AED in seizure history, cortical entry, or high‑risk tumors per SOP; no blanket prophylaxis for all meningiomas/schwannomas. | Drug/endpoints per treating team; documentation required if deviating. |
| **Steroid policy** | Dexamethasone for vasogenic edema/symptomatic mass effect; taper post‑op guided by neuro status and imaging. | Stress‑ulcer and glucose monitoring per SOP. |
| **DVT/VTE prevention** | Mechanical prophylaxis on admission; pharmacologic prophylaxis (LMWH or UFH) post‑op when hemostasis secured and no contraindication. | Restart/hold in anticoagulated patients per SOP with neurosurgical and anesthetic input. |
| **Intraoperative adjuncts** | Neuronavigation and ultrasound routinely available; IONM and awake mapping in eloquent‑area surgery when indicated; 5‑ALA use per glioma protocol. | Microscope/endoscope selection per pathology and approach. |
| **Extubation & immediate surveillance** | Extubation in OR/PACU when criteria met; standardized neuro checks (e.g., every 30–60 min initially, then spaced). | Failure to meet criteria triggers IMC/ICU transfer. |
| **Triage: ICU/IMC vs ward** | **Criteria‑based**, not routine ICU admission. Triggers include any of: emergency surgery; infratentorial/posterior fossa cases with mass effect or hydrocephalus; EVD/ICP device in situ; significant intraoperative events (e.g., blood loss, air embolism, new neuro deficit); GCS ≤ 13 on emergence; need for vasoactive/respiratory support; major comorbidity (e.g., ASA ≥ 3 with instability). | Aligns with our prior discussion advocating selective ICU use rather than routine admission. 00.00.00 00:00:00 |
| **Early postoperative imaging** | CT within 24 h after supratentorial craniotomy or as clinically indicated; early MRI (protocol‑specific) for glioma/meningioma extent‑of‑resection assessment per tumor pathway. | Imaging timing may be advanced if neurological change occurs. |
| **Mobilization & swallow** | Day‑0/Day‑1 mobilization if stable; swallow screen before oral intake when indicated; standardized pain and PONV prophylaxis. | Escalation to physiotherapy/speech therapy per findings. |
| **Wound care** | Sterile dressing protocol; drain management per SOP; suture/staple removal timelines documented. | CSF leak algorithm activated on suspicion. |
| **AE capture & review** | POPAE form completed at discharge, reviewed by senior attending; automatic 30‑day readmission flagging; MMC presentation for complex cases; AE grading by Clavien–Dindo. | Program and definitions per Dao Trong et al., 2023; applied to the intracranial tumor cohort as in Lenga et al., 2024. [PMC+1](https://pmc.ncbi.nlm.nih.gov/articles/PMC10006024/) |

**Abbreviations:** AE, adverse event; AED, antiepileptic drug; ASA, American Society of Anesthesiologists; CT, computed tomography; EBL, estimated blood loss; EVD, external ventricular drain; GCS, Glasgow Coma Scale; ICU, intensive care unit; IMC, intermediate care unit; IONM, intraoperative neurophysiological monitoring; LMWH, low‑molecular‑weight heparin; MMC, morbidity and mortality conference; PACU, post‑anesthesia care unit; POPAE, postoperative adverse‑event evaluation; SOP, standard operating procedure; UFH, unfractionated heparin.
